# Uric Acid-Driven Biomarkers and Clinical Outcomes in Metastatic Pancreatic Cancer: A Multicenter Real-World Cohort Study

**DOI:** 10.3390/diagnostics16091296

**Published:** 2026-04-26

**Authors:** Ahmet Unlu, Asim Armagan Aydin, Mehmet Nuri Baser, Merve Turan, Murat Kocer, Banu Ozturk, Mustafa Yildiz

**Affiliations:** 1 Department of Medical Oncology, University of Health Sciences, Antalya Training and Research Hospital, 07100 Antalya, Turkey; drarmaganaydin@gmail.com (A.A.A.); muratkocer71@hotmail.com (M.K.); drbanutr@yahoo.com (B.O.); drmyildiz@yahoo.com (M.Y.); 2 Department of Medical Oncology, Faculty of Medicine, Aydin Adnan Menderes University, 09100 Aydin, Turkey; mehmetnuribaser@gmail.com (M.N.B.); drmerveturan@gmail.com (M.T.)

**Keywords:** uric acid to albumin ratio, uric acid to lymphocyte ratio, uric acid, pancreatic cancer, advanced stage, albumin, inflammation and cancer, chemotherapy failure, prognosis, survival

## Abstract

**Background/Objectives**: Metastatic pancreatic cancer is a highly lethal disease, and clinically useful biomarkers for outcome stratification are limited. Uric acid reflects systemic metabolic stress and inflammatory signaling, suggesting potential relevance as a tumor–host biomarker. However, the clinical significance of uric acid-based composite biomarkers in pancreatic cancer remains unclear. **Methods**: In this multicenter retrospective cohort study, 110 patients with metastatic pancreatic adenocarcinoma treated between 2015 and 2024 were analyzed. Sex-adjusted uric acid-based biomarkers were calculated using uric acid z-scores normalized by sex and integrated with markers of nutritional and immune status, including the uric acid z-score-to-albumin ratio (UAzAR) and uric acid z-score-to-lymphocyte ratio (UAzLR). Associations with overall survival (OS), progression-free survival (PFS), and chemotherapy response were evaluated using Kaplan–Meier analysis, Cox proportional hazards models, receiver operating characteristic (ROC) analyses, and multivariate logistic regression. **Results**: The median OS and PFS for the entire cohort were 12.6 months (95% CI 11.3–13.9) and 7.5 months (95% CI 6.6–8.4), respectively. Patients with high UAzAR had significantly shorter OS than those with low UAzAR (7.3 vs. 16.4 months; log-rank *p* < 0.001), and similar findings were observed for UAzLR (7.4 vs. 16.4 months; *p* < 0.001). In multivariate Cox models, elevated UAzAR independently predicted inferior OS (HR] 3.10, 95% CI 1.58–6.09; *p* = 0.001) and PFS (HR 2.35, 95% CI 1.22–4.52; *p* = 0.010), while elevated UAzLR was similarly associated with reduced OS (HR 3.28, 95% CI 1.68–6.39; *p* < 0.001) and PFS (HR 2.47, 95% CI 1.30–4.70; *p* = 0.006). High UAzAR and UAzLR were also independently associated with chemotherapy failure (adjusted odds ratio [OR] 5.52, 95% CI 2.16–14.06 and OR 6.42, 95% CI 2.49–16.55; both *p* < 0.001). In ROC analyses, UAzAR and UAzLR demonstrated moderate discrimination for 12-month OS (AUC 0.659 and 0.658) and stronger discrimination for 6-month PFS (AUC 0.705 and 0.692). **Conclusions**: Sex-adjusted uric acid-derived composite biomarkers independently predict survival and chemotherapy response in metastatic pancreatic cancer and may identify a high-risk metabolic phenotype relevant for clinical risk stratification.

## 1. Introduction

The pancreatic cancer remains one of the most lethal malignancies worldwide and is projected to become a leading cause of cancer-related mortality in the coming decades [1]. According to recent GLOBOCAN estimates, more than 495,000 new cases and approximately 466,000 deaths occur annually, underscoring the aggressive biology and limited therapeutic responsiveness of this disease [2]. Despite incremental advances in systemic therapy—including multi-agent regimens such as FOLFIRINOX, gemcitabine-based combinations, and more recently, liposomal irinotecan-based and NALIRIFOX strategies—clinical outcomes remain poor, with median overall survival (OS) in metastatic disease rarely exceeding one year [3,4]. Moreover, treatment responses remain highly heterogeneous among patients with otherwise comparable clinicopathological characteristics, highlighting a fundamental limitation of current risk-stratification approaches [5].

Growing evidence indicates that systemic immune, nutritional, and metabolic states play a critical role in shaping cancer outcomes [6,7]. Biomarkers reflecting host inflammatory burden and physiological reserve—such as the neutrophil-to-lymphocyte ratio (NLR), albumin-based indices, and other composite immune–nutrition scores—have consistently demonstrated prognostic value across multiple malignancies, including pancreatic cancer [8,9,10,11]. These markers reflect a broader biological framework in which systemic inflammation, metabolic stress, and immune dysfunction converge to influence tumor progression and treatment tolerance [12,13]. Within this context, uric acid is a biologically intriguing candidate biomarker [14,15]. As the terminal product of purine metabolism, uric acid reflects heightened cellular turnover, oxidative stress, and metabolic dysregulation—features characteristic of rapidly proliferating malignancies, such as pancreatic cancer [16,17,18]. In addition to its metabolic implications, uric acid can function as a damage-associated molecular pattern (DAMP), capable of modulating innate immune signaling, inflammatory cascades, and lymphocyte function [19,20]. Mechanistically, uric acid has been shown to activate innate immune pathways through (NOD-, LRR- and pyrin domain-containing protein 3 (NLRP3) inflammasome signaling, leading to the production of pro-inflammatory cytokines such as interleukin-1β and thereby amplifying sterile inflammatory responses [21,22]. In parallel, soluble uric acid contributes to neutrophil recruitment and adhesion via β2-integrin–dependent pathways, shaping early innate immune dynamics. Beyond these immediate effects, uric acid–driven signaling has been implicated in macrophage activation and dendritic cell maturation, linking innate sensing to adaptive immune priming [23]. Importantly, this immunomodulatory axis appears context-dependent: while acute activation may support host defense, chronic uric acid elevation may sustain low-grade inflammation, promote immune dysregulation, and foster a tumor-permissive microenvironment. Collectively, these features position uric acid as a biologically plausible integrator of metabolic stress and immune signaling within the tumor–host interface, with potential implications for risk stratification and therapeutic vulnerability in cancer. Prior clinical studies on various cancer types have reported associations between elevated uric acid levels and adverse oncologic outcomes, suggesting that uric acid may capture a host metabolic phenotype linked to aggressive disease biology and impaired therapeutic responsiveness [24,25,26].

However, the clinical significance of uric acid-based biomarkers in pancreatic cancer remains incompletely defined. Interpretation is further complicated by substantial physiological variability in circulating uric acid levels between men and women, largely driven by hormonal regulation and differences in renal urate handling [27,28]. Accounting for this biological heterogeneity may therefore be essential for uncovering clinically meaningful associations [14,29]. Integrating sex-adjusted uric acid metrics with established markers of nutritional and immune status—such as albumin and lymphocyte counts—may provide a more biologically coherent representation of host metabolic vulnerability and improve risk stratification in advanced pancreatic cancer.

In this multicenter real-world cohort study, we investigated the prognostic and predictive significance of sex-adjusted uric acid-derived composite biomarkers in patients with metastatic pancreatic cancer. Specifically, we evaluated the uric acid z-score-to-albumin ratio (UAzAR) and the uric acid z-score-to-lymphocyte ratio (UAzLR) as integrative markers of metabolic, nutritional, and immune stress and examined their associations with survival outcomes and response to first-line chemotherapy.

## 2. Materials and Methods

### 2.1. Study Design and Setting

This multicenter, real-world, retrospective cohort study was conducted in accordance with the Strengthening the Reporting of Observational Studies in Epidemiology (STROBE) recommendations. Adult patients newly diagnosed with pancreatic cancer between January 2015 and December 2024 were retrospectively identified from participating centers, including the University of Health Sciences Antalya Training and Research Hospital and the Aydın Adnan Menderes University Faculty of Medicine.

**Figure 1:**
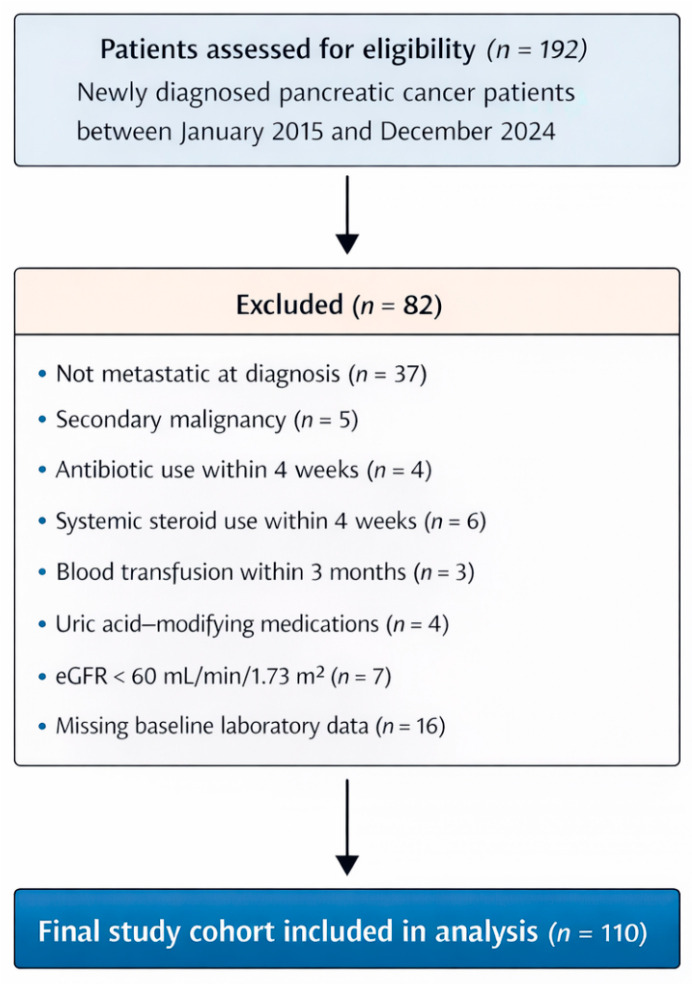
Flow diagram of patient selection and final study cohort.

A total of 192 patients were initially assessed for eligibility. After application of predefined inclusion and exclusion criteria, 110 patients with metastatic pancreatic adenocarcinoma comprised the final study cohort included in the analysis (Figure 1).

The study was conducted in accordance with the Declaration of Helsinki and was approved by the Institutional Review Board of the University of Health Sciences Antalya Training and Research Hospital and the Antalya Provincial Health Directorate (approval no. 2/15; 22 January 2026). The requirement for informed consent was waived due to the retrospective design and use of anonymized data.

### 2.2. Study Population

The study population comprised adult patients (≥18 years) with histopathologically confirmed metastatic pancreatic adenocarcinoma. Patients were eligible if comprehensive clinical, laboratory, pathological, and follow-up data required for reliable survival analyses were available and if a minimum clinical and radiological follow-up of six months was documented, unless death occurred prior to this threshold.

Of the initially screened 192 patients, 82 were excluded for the following reasons: absence of metastatic disease at diagnosis (*n* = 37), presence of a second active malignancy (*n* = 5), recent antibiotic use within 4 weeks (*n* = 4), systemic steroid use within 4 weeks (*n* = 6), blood transfusion within 3 months (*n* = 3), use of uric acid-modifying medications (*n* = 4), impaired renal function defined as eGFR < 60 mL/min/1.73 m^2^ (*n* = 7), and missing baseline laboratory data (*n* = 16). The remaining 110 patients constituted the final study cohort for all survival and treatment-response analyses (Figure 1). To minimize potential confounding effects on systemic inflammatory and metabolic parameters—particularly serum uric acid levels—these exclusion criteria were specifically designed to reduce the influence of acute inflammatory conditions, metabolic disturbances, and medications known to affect uric acid homeostasis, thereby ensuring a more reliable assessment of baseline biomarker status.

### 2.3. Data Collection

Clinical, pathological, treatment-related, and laboratory data were retrospectively collected from the electronic medical records of participating centers using a standardized data collection template. Each center contributed anonymized data according to predefined study variables to ensure consistency across institutions.

The collected variables included demographic characteristics (age at diagnosis and sex), clinical features (Eastern Cooperative Oncology Group performance status, smoking status, diabetes mellitus, alcohol use, obesity, and comorbid conditions), and tumor-related parameters (primary tumor location and sites of metastatic involvement, including the liver, lung, and peritoneum). Treatment-related variables comprised the type of first-line systemic chemotherapy regimen and the best radiological response to treatment.

Baseline laboratory assessments were performed using peripheral venous blood samples collected under routine clinical conditions within 7 days prior to the initiation of first-line systemic therapy to ensure a standardized pretreatment evaluation. Serum biochemical parameters, including uric acid, albumin, lactate dehydrogenase (LDH), and C-reactive protein (CRP), were measured using automated clinical chemistry analyzers, while complete blood count components (neutrophils, lymphocytes, monocytes, and platelets) were obtained using standardized hematology analyzers. All samples were processed in accordance with institutional laboratory protocols at each participating center, with adherence to internal quality control procedures to ensure analytical consistency across sites.

### 2.4. Biomarker Construction and Definitions

Biomarker analyses were based on baseline laboratory parameters obtained prior to the initiation of systemic therapy. To account for physiological sex-related variability in serum uric acid levels, sex-specific standardization was performed using z-score transformation. The uric acid z-score (UA_z) was calculated as follows:

UA_z = (UA_i − μ_{sex})/σ_{sex}

where UA_i represents an individual patient’s serum uric acid level, and μ_{sex} and σ_{sex} denote the sex-specific mean and standard deviation within the study cohort. This approach was applied to enable biologically comparable interpretation of uric acid levels across patients.

Sex-adjusted uric acid-based composite biomarkers were derived by integrating UA_z with markers of nutritional and immune status. The UAzAR was calculated as UA_z divided by serum albumin (g/dL), and the UAzLR was calculated as UA_z divided by absolute lymphocyte count (×10^9^/L).

These composite indices reflect an integrated host phenotype encompassing metabolic stress (UAzAR), nutritional reserve (albumin), and immune competence (lymphocyte count). Higher values of UAzAR and UAzLR indicate a relatively elevated metabolic burden in the context of impaired host reserve.

Optimal cut-off values for UAzAR and UAzLR were determined using receiver operating characteristic (ROC) curve analysis for 12-month overall survival, based on the maximization of the Youden index. The derived cut-offs (UAzAR: 0.0102; UAzLR: 0.00026) were subsequently applied in all survival and treatment-response analyses.

For comparative purposes, established inflammatory and metabolic indices were also calculated from baseline laboratory parameters, including NLR [8], C-reactive protein-to-albumin ratio (CAR) [9], lactate dehydrogenase-to-albumin ratio (LAR) [30], and the global immune-nutrition-inflammation index (GINI) [31], using standard formulas.

### 2.5. Treatment Protocols and Clinical Follow-Up

Patients received standard-of-care first-line systemic therapy for metastatic pancreatic adenocarcinoma in accordance with institutional protocols and contemporary clinical guidelines. First-line treatment predominantly consisted of combination chemotherapy regimens, including FOLFIRINOX, nab-paclitaxel plus gemcitabine, or platinum-based regimens with gemcitabine. No patients in this cohort received immunotherapy as part of first-line treatment, reflecting the limited role of immune checkpoint inhibitors in routine clinical practice for metastatic pancreatic cancer during the study period. Treatment selection was based on patient-related clinical characteristics, performance status, comorbidities, and the physician’s discretion. Clinical follow-up was conducted through routine outpatient evaluation and radiological imaging, with disease progression defined by radiological evidence and/or clinical deterioration.

Tumor response to first-line therapy was evaluated using radiological assessments and categorized as complete response (CR), partial response (PR), stable disease (SD), or progressive disease (PD), interpreted in accordance with the Response Evaluation Criteria in Solid Tumors (RECIST) version 1.1. For analytical purposes, treatment response was further dichotomized as disease control (CR, PR, or SD) versus PD.

Progression-free survival (PFS) was defined as the time from the date of diagnosis of metastatic disease to the first documented disease progression, death from any cause, or last follow-up, whichever occurred first. OS was defined as the time from the date of diagnosis of metastatic disease to death from any cause or last follow-up. Patients without events were censored at the date of the last clinical contact.

### 2.6. Statistical Analysis

All statistical analyses were performed using R statistical software (version 4.3.1; R Foundation for Statistical Computing, Vienna, Austria). Continuous variables are presented as medians and interquartile ranges (IQR), and categorical variables as frequencies and percentages. Group comparisons were conducted using the Mann–Whitney U test for continuous variables and the χ^2^ test or Fisher’s exact test for categorical variables, as appropriate. OS and PFS were estimated using the Kaplan–Meier method and compared using the log-rank test. The median follow-up time was calculated using the reverse Kaplan–Meier method.

Associations with OS and PFS were first evaluated using univariate Cox proportional hazards regression analyses. Subsequently, multivariate Cox regression models were constructed to identify independent prognostic factors. Variables with a univariate *p*-value < 0.10 were considered for inclusion, whereas age and ECOG performance status were forced into all models based on clinical relevance. To avoid collinearity, UAzAR and UAzLR were evaluated in separate multivariate models. Hazard ratios (HRs) and corresponding 95% confidence intervals (CIs) are reported. The proportional hazards assumption was assessed using Schoenfeld residuals.

The discriminative performance of pretreatment inflammatory and metabolic indices was evaluated using receiver operating characteristic (ROC) curve analyses. All ROC analyses were based on pretreatment biomarker values, thereby reflecting baseline risk stratification. Clinically predefined time points (12-month OS and 6-month PFS) were selected to evaluate discriminative performance within meaningful and interpretable outcome horizons, capturing both early disease progression and clinically relevant survival outcomes. Classical ROC analyses were performed with optimal cut-off values determined by maximizing the Youden index. Comparisons of area under the curve (AUC) values were conducted using DeLong’s test. Time-dependent ROC analyses were performed using inverse probability of censoring weighting (IPCW) to assess predictive performance over time.

Logistic regression analyses were performed to evaluate the predictors of chemotherapy response. Chemotherapy failure was defined as progressive disease as the best response to first-line therapy. Multivariable logistic regression models were adjusted for age (≥65 years), ECOG performance status (0–1 vs. ≥2), presence of liver metastasis, and first-line chemotherapy regimen (FOLFIRINOX vs. non-FOLFIRINOX). Ordinal logistic regression analyses were also performed to assess the associations between uric acid-based indices and ordered treatment response categories (CR, PR, SD, and PD). All statistical tests were two-sided, and a *p*-value < 0.05 was considered statistically significant.

## 3. Results

### 3.1. Baseline Characteristics of the Study Cohort

A total of 110 patients with metastatic pancreatic cancer were included in the final analysis. The median age was 65 years (IQR, 57–70), and 58 patients (52.7%) were aged ≥65 years. The cohort was predominantly male (69/110, 62.7%). Most patients had an ECOG performance status of 0–1 at diagnosis (76 patients, 69.1%). Lifestyle- and comorbidity-related factors were common, including smoking (57/110, 51.8%), comorbid conditions (57/110, 51.8%), and diabetes mellitus (35/110, 31.8%), whereas alcoholism (24/110, 21.8%) and obesity (11/110, 10.0%) were less frequent. In terms of disease characteristics, the primary tumor was most commonly located in the pancreatic head (65/110, 59.1%), followed by the body (23/110, 20.9%) and tail (22/110, 20.0%). At diagnosis, liver metastasis was present in 76 patients (69.1%), while lung metastasis and peritoneal involvement were observed in 15 (13.6%) and 26 (23.6%) patients, respectively. Regarding first-line systemic therapy, FOLFIRINOX was administered to 65 patients (59.1%), nab-paclitaxel-based therapy to 16 (14.5%), and platinum plus gemcitabine regimens to 13 (11.8%), while 16 patients (14.5%) received other regimens. Disease control following first-line chemotherapy was achieved in 71 patients (64.5%).

Treatment response rates were further analyzed according to metastatic site. Among patients with liver metastasis, disease control was achieved in 63.2% of cases, compared with 67.6% in those without liver involvement (*p* = 0.67). Similarly, disease control rates were 60.0% in patients with lung metastasis and 65.3% in those without (*p* = 0.77). For peritoneal involvement, disease control was observed in 65.4% of patients with peritoneal metastasis and 64.3% of those without (*p* = 1.00). Overall, treatment response rates were comparable across metastatic subgroups, with no statistically significant differences observed.

Baseline clinical, pathological, and treatment-related characteristics stratified by sex-adjusted uric acid-based indices are summarized in Table 1.

### 3.2. Survival Analysis

During the follow-up of the overall cohort (*n* = 110), disease progression occurred in 109 patients (99.1%), and 103 patients (93.6%) died. The median follow-up duration, estimated using the reverse Kaplan–Meier method, was 46.0 months (95% CI, 39.2–52.8). The median OS for the entire cohort was 12.6 months (95% CI, 11.3–13.9), while the median PFS was 7.5 months (95% CI, 6.6–8.4).

**Figure 2:**
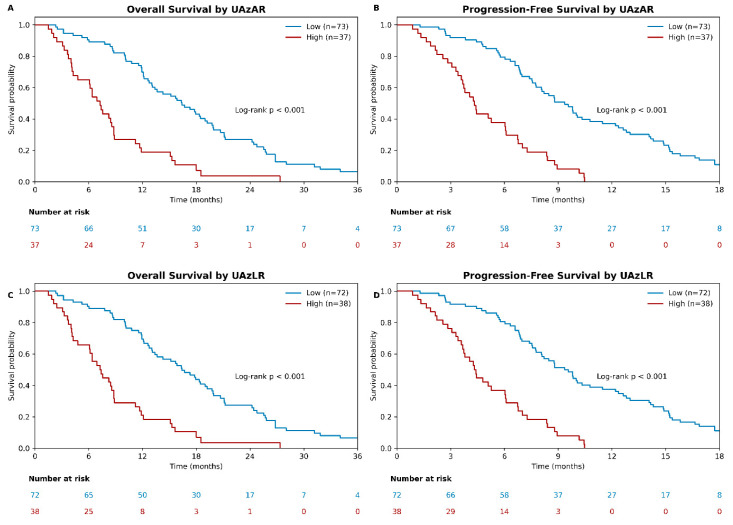
Kaplan–Meier survival curves according to sex-adjusted uric acid-based indices. Kaplan–Meier curves show overall survival (OS) and progression-free survival (PFS) stratified by sex-adjusted uric acid-to-albumin ratio (UAzAR) and uric acid-to-lymphocyte ratio (UAzLR). Panels display OS and PFS according to UAzAR (**A**,**B**) and UAzLR (**C**,**D**). Survival distributions differed significantly between the low- and high-index groups, as assessed by the log-rank test. Numbers at risk are provided below the time axis. Cut-off values were defined using Youden index-based optimization.

Kaplan–Meier analyses showed a clear separation of survival curves according to sex-adjusted uric acid-based indices. For UAzAR, patients in the low group had longer OS than those in the high group (16.4 months [95% CI, 14.2–18.6] vs. 7.3 months [95% CI, 6.1–8.5]; log-rank *p* < 0.001). Similarly, for UAzLR, the median OS was 16.4 months (95% CI, 14.1–18.7) in the low group and 7.4 months (95% CI, 6.2–8.6) in the high group (log-rank *p* < 0.001) (Figure 2).

Comparable findings were observed for PFS. Patients with low UAzAR experienced a longer median PFS than those with high UAzAR (9.4 months [95% CI, 8.3–10.5] vs. 4.3 months [95% CI, 3.6–5.0]; log-rank *p* < 0.001). Likewise, low UAzLR was associated with prolonged PFS compared with high UAzLR (9.4 months [95% CI, 8.2–10.6] vs. 4.4 months [95% CI, 3.7–5.1]; log-rank *p* < 0.001) (Figure 2).

### 3.3. Discriminative Performance of Sex-Adjusted Uric Acid-Based İndices

**Figure 3:**
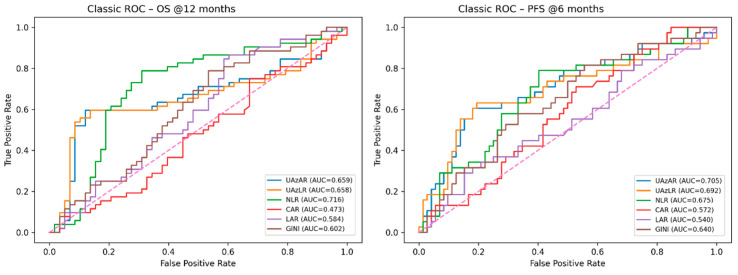
Classical ROC curves for survival outcomes. Classical ROC curves illustrate the discriminative performance of pretreatment indices for 12-month overall survival (OS) and 6-month progression-free survival (PFS). The curves are shown for the sex-adjusted uric acid-to-albumin ratio (UAzAR), the uric acid-to-lymphocyte ratio (UAzLR), and comparator inflammatory indices. Area under the curve (AUC) values with corresponding *p* values are reported. Optimal cutoff values were determined using the Youden index.

The discriminative ability of pretreatment inflammatory and metabolic indices for survival outcomes was evaluated using receiver operating characteristic analyses. Classical receiver operating characteristic analyses were performed for 12-month OS and 6-month PFS, with optimal cut-off values determined using the Youden index (Figure 3).

In classical ROC analyses, UAzAR and UAzLR demonstrated comparable discriminative performance for 12-month OS, with AUC values of 0.659 and 0.658, respectively (both *p* < 0.001) (Figure 3). For 6-month PFS, the discriminative performance of both indices was numerically higher, with AUC values of 0.705 for UAzAR and 0.692 for UAzLR (both *p* < 0.001). The Youden index-derived cutoff values were 0.0102 for UAzAR and 0.00026 for UAzLR, yielding high specificity for OS prediction (0.879 and 0.862, respectively) (Figure 3, Table 2).

Comparative analyses with established inflammatory markers showed that NLR achieved the highest AUC for 12-month OS, whereas UAzAR and UAzLR demonstrated more balanced discriminative profiles for early PFS, with higher or comparable AUC values relative to CAR, LAR, and GINI (Table 2). In pairwise comparisons using DeLong’s test, no statistically significant differences in AUC values were observed between UAzAR and NLR or between UAzLR and NLR for either 12-month OS or 6-month PFS (all *p* > 0.05).

**Figure 4:**
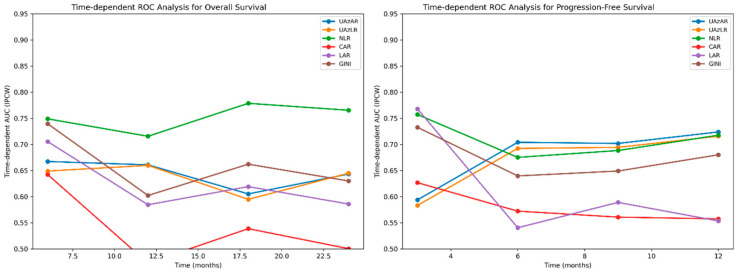
Time-dependent ROC analyses for overall survival and progression-free survival. Time-dependent receiver operating characteristic (ROC) curves based on inverse probability of censoring weighting depict the discriminative performance of pretreatment indices for overall survival (OS) and progression-free survival (PFS) at multiple time points. AUC values are presented for sex-adjusted uric acid-based indices and comparator inflammatory markers, demonstrating changes in discriminative ability over time.

To account for censoring and evaluate discriminative performance over time, time-dependent ROC analyses were conducted using inverse probability of censoring weighting (IPCW; Figure 4). For OS, UAzAR and UAzLR showed stable, moderate discriminative performance across clinically relevant time points (6–24 months), with AUC values remaining consistently above 0.60. In PFS analyses, both indices demonstrated sustained discriminative ability across all evaluated time points, with peak performance observed between 6 and 12 months, during which UAzAR and UAzLR consistently ranked among the highest-performing indices (Figure 4). Numerical AUC values from the time-dependent ROC analyses are provided in the Supplementary Materials.

### 3.4. Association of UAzAR and UAzLR with Chemotherapy Response

Treatment response to first-line chemotherapy was evaluated to examine the association between sex-adjusted uric acid-based indices and treatment efficacy. Using a binary response definition (any response [CR/PR/SD] versus no response [PD]), patients with elevated UAzAR and UAzLR values exhibited significantly lower response rates. Chemotherapy failure was observed more frequently among patients with high UAzAR (62.2% vs. 21.9%) and high UAzLR (63.2% vs. 20.8%) compared with those in the corresponding low-index groups (both *p* < 0.001).

After adjustment for age, ECOG performance status, presence of liver metastasis at diagnosis, and first-line chemotherapy regimen, both indices remained independently associated with chemotherapy failure. High UAzAR was associated with a 5.52-fold increased odds of no response (95% CI, 2.16–14.06; *p* < 0.001), while high UAzLR conferred a 6.42-fold increased odds of treatment failure (95% CI, 2.49–16.55; *p* < 0.001) (Table 3).

**Figure 5:**
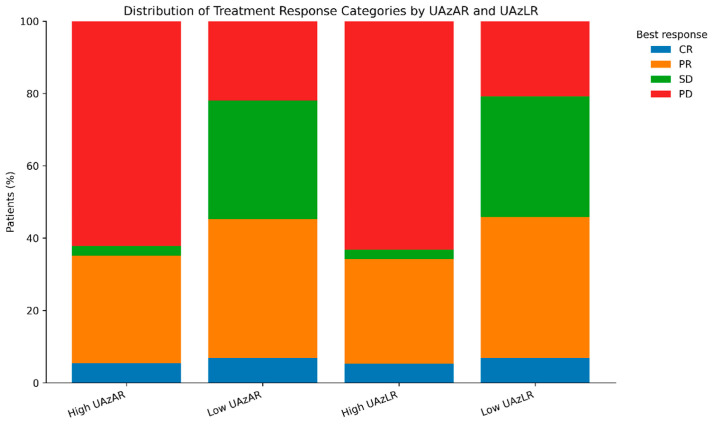
Distribution of first-line chemotherapy response categories according to sex-adjusted uric acid-based indices. Stacked bar plots depict the distribution of best responses to first-line chemotherapy in the low- and high-UAzAR and UAzLR groups. Response categories included complete response (CR), partial response (PR), stable disease (SD), and progressive disease (PD). Differences in response distributions between the index strata are illustrated.

Consistent results were observed in analyses based on the four-category response classification (CR, PR, SD, and PD). Ordinal logistic regression demonstrated that elevated UAzAR (adjusted common OR, 2.78; 95% CI, 1.21–6.37; *p* = 0.016) and UAzLR (adjusted common OR, 3.08; 95% CI, 1.34–7.05; *p* = 0.0079) were independently associated with worsening response categories. The distribution of treatment response categories across UAzAR and UAzLR strata is shown in Figure 5.

### 3.5. Univariate and Multivariable Survival Analyses

Univariate Cox regression analyses were performed for a comprehensive set of demographic, clinical, and laboratory variables, identifying age, performance status, systemic inflammatory indices, treatment response, and sex-adjusted uric acid-based indices as factors associated with OS and PFS (Table 4).

In multivariable Cox regression models adjusted for clinically relevant covariates, both UAzAR and UAzLR retained independent associations with survival outcomes. When included in separate models to avoid collinearity, high UAzAR was independently associated with inferior OS (HR, 3.10; 95% CI, 1.58–6.09; *p* = 0.001) and PFS (HR, 2.35; 95% CI, 1.22–4.52; *p* = 0.010). Similarly, high UAzLR remained independently associated with reduced OS (HR, 3.28; 95% CI, 1.68–6.39; *p* < 0.001) and PFS (HR, 2.47; 95% CI, 1.30–4.70; *p* = 0.006) (Table 5).

Across all models, treatment response to first-line chemotherapy was consistently associated with improved OS and PFS, whereas elevated NLR and non-FOLFIRINOX regimens were independently associated with inferior outcomes in selected models (Table 5).

## 4. Discussion

In this multicenter real-world cohort of patients with metastatic pancreatic cancer, we demonstrated that sex-adjusted uric acid–driven composite biomarkers capture a clinically meaningful tumor–host phenotype characterized by metabolic stress, immune dysfunction, and reduced treatment responsiveness. Both UAzAR and UAzLR consistently stratified patients according to overall and progression-free survival and independently predicted failure of first-line chemotherapy. These findings extend beyond conventional prognostic associations and support a biologically integrated framework in which uric acid–related pathways reflect not only disease burden but also host vulnerability to systemic therapy.

A key strength of the present study is the consistency of these associations across complementary analytical approaches. Uric acid-based indices demonstrated robust separation of Kaplan–Meier survival curves, retained independent prognostic significance in multivariate models, and exhibited stable discriminative performance across both classical and time-dependent ROC analyses. Notably, their predictive value extended to early disease progression and treatment response, suggesting that these biomarkers capture dynamic aspects of disease behavior that are not fully encompassed by traditional clinicopathological factors. Together, these findings suggest that uric acid-based indices capture biologically meaningful processes underlying disease progression.

From a translational perspective, uric acid represents a clinically accessible surrogate of the tumor–host interface, integrating signals related to metabolic stress, systemic inflammation, and host physiological reserve [14,17]. While its mechanistic links to innate immune activation and inflammatory signaling have been established in prior studies [19,32], the clinical relevance of uric acid in oncology likely extends beyond isolated biological pathways. Rather, it reflects a broader host phenotype shaped by tumor-driven metabolic demand and immune–nutritional status. In this context, the integration of uric acid with albumin and lymphocyte counts (UAzAR and UAzLR) provides a composite framework that captures three interdependent domains: metabolic burden, nutritional reserve, and immune competence. These dimensions are increasingly recognized as key determinants of treatment tolerance, early disease progression, and survival heterogeneity in metastatic pancreatic cancer [16,33]. Accordingly, our findings suggest that uric acid-based composite indices may serve as pragmatic tools for baseline risk stratification, offering clinically relevant insight beyond conventional inflammation-based markers alone.

The observed association between elevated UAzAR and UAzLR values and chemotherapy failure provides important clinical insights. Chemoresistance in pancreatic cancer is increasingly recognized as a multifactorial process influenced not only by tumor-intrinsic mechanisms but also by host-related factors, including systemic inflammation, metabolic stress, and nutritional depletion [34,35]. Patients characterized by high uric acid-based indices may therefore represent a biologically vulnerable subgroup in whom impaired host reserve and dysregulated immune–metabolic signaling limit effective antitumor responses. The persistence of these associations after adjustment for performance status, metastatic burden, and treatment regimen suggests that uric acid–driven host biology captures clinically actionable information beyond conventional prognostic markers. In this context, the presence of liver metastasis may further contribute to treatment resistance through distinct biological mechanisms. The liver represents a unique immunologically tolerant microenvironment that may attenuate effective antitumor immune responses [36]. In addition, higher tumor burden, altered hepatic drug metabolism, and amplified systemic inflammatory signaling may collectively reduce chemotherapy efficacy in this subgroup [37]. These mechanisms may partly explain the biological context in which elevated uric acid-based indices identify patients with increased susceptibility to treatment failure.

These findings have potential implications for clinical practice. The early identification of patients at a high risk of treatment failure remains a critical unmet need in metastatic pancreatic cancer. Uric acid-based composite biomarkers may serve as accessible tools to refine baseline risk stratification and identify patients unlikely to benefit from standard first-line chemotherapy. In such patients, alternative therapeutic strategies, closer clinical monitoring, or early treatment modification could be considered. Although prospective validation is required, the integration of metabolic–immune biomarkers into clinical decision-making frameworks may represent an important step toward more individualized treatment approaches for this disease.

Importantly, while established inflammatory indices, such as the neutrophil-to-lymphocyte ratio, have demonstrated strong prognostic performance [8], uric acid-based biomarkers offer a more integrative representation of host biology. By simultaneously capturing metabolic stress, nutritional status, and immune competence, UAzAR and UAzLR reflect complementary biological dimensions that are not fully encompassed by traditional inflammatory markers alone. The comparable discriminative performance observed across these indices suggests that uric acid-derived composites are likely to complement, rather than replace, existing biomarker strategies, particularly in identifying patients at risk of early disease progression within the first 6 months of treatment, as reflected by PFS-based analyses, and in capturing vulnerability to treatment resistance.

Several limitations should be acknowledged. The predominance of male patients in this cohort reflects known epidemiological patterns in pancreatic cancer; however, given the well-established sex-related variability in serum uric acid levels, this imbalance represents a potential source of bias. To address this, we applied sex-specific standardization using z-score transformation, aiming to ensure biologically meaningful comparability across patients. Given that all patients in this study had metastatic (stage IV) disease at diagnosis, the additional prognostic contribution of formal TNM staging was considered limited. Instead, variables reflecting metastatic burden and distribution were incorporated as more clinically relevant indicators of disease extent in this setting. The retrospective design and real-world treatment heterogeneity introduce the potential for unmeasured confounding despite multivariable adjustment. Although sex-adjusted normalization was applied to mitigate physiological variability in uric acid levels, residual confounding related to renal function, comorbid conditions, or concomitant medications cannot be fully excluded. In addition, the lack of longitudinal biomarker assessment precludes the evaluation of dynamic changes in uric acid-based indices during treatment. External validation in independent cohorts was not available. Nevertheless, the multicenter design, consistency of findings across multiple analytical approaches, and integration of survival and treatment-response outcomes strengthen the robustness and clinical relevance of our results.

## 5. Conclusions

Sex-adjusted uric acid-driven composite biomarkers represent robust and biologically grounded predictors of survival and chemotherapy response in metastatic pancreatic cancer. By integrating tumor-associated metabolic stress with host nutritional and immune reserves, UAzAR and UAzLR define a clinically meaningful high-risk phenotype that is not fully captured by conventional inflammatory indices. These findings support the incorporation of uric acid-based biomarkers into prognostic frameworks and highlight the potential of tumor–host metabolic profiling to refine risk stratification and inform treatment decision-making. Prospective validation is warranted to determine their role in biomarker-guided therapeutic strategies for advanced pancreatic cancer.

## Figures and Tables

**Table 1:** Baseline characteristics of the cohort stratified by sex-adjusted uric acid-based indices (*n* = 110).

Characteristic		Uric Acid-to-Albumin Ratio (UAzAR), *n* (%)			Uric Acid-to-Lymphocyte Ratio (UAzLR), *n* (%)		
		Low	High	*p*	Low	High	*p*
Age	<65	40 (54.8)	12 (32.4)	0.044	40 (55.6)	12 (31.6)	0.028
	≥65	33 (45.2)	25 (67.6)		32 (44.4)	26 (68.4)	
Sex	Female	28 (38.4)	13 (35.1)	0.903	28 (38.9)	13 (34.2)	0.783
	Male	45 (61.6)	24 (64.9)		44 (61.1)	25 (65.8)	
ECOG	0–1	50 (68.5)	26 (70.3)	1.000	49 (68.1)	27 (71.1)	0.915
	≥2	23 (31.5)	11 (29.7)		23 (31.9)	11 (28.9)	
Alcoholism	No	59 (80.8)	27 (73.0)	0.486	58 (80.6)	28 (73.7)	0.557
	Yes	14 (19.2)	10 (27.0)		14 (19.4)	10 (26.3)	
Smoking	No	36 (49.3)	17 (45.9)	0.895	36 (50.0)	17 (44.7)	0.745
	Yes	37 (50.7)	20 (54.1)		36 (50.0)	21 (55.3)	
Obesity	No	67 (91.8)	32 (86.5)	0.590	66 (91.7)	33 (86.8)	0.640
	Yes	6 (8.2)	5 (13.5)		6 (8.3)	5 (13.2)	
Diabetes	No	52 (71.2)	23 (62.2)	0.454	51 (70.8)	24 (63.2)	0.544
	Yes	21 (28.8)	14 (37.8)		21 (29.2)	14 (36.8)	
Comorbidity	No	37 (50.7)	16 (43.2)	0.592	36 (50.0)	17 (44.7)	0.745
	Yes	36 (49.3)	21 (56.8)		36 (50.0)	21 (55.3)	
Anatomic tumor location	Head	45 (61.6)	20 (54.1)	0.258	44 (61.1)	21 (55.3)	0.293
	Body	12 (16.4)	11 (29.7)		12 (16.7)	11 (28.9)	
	Tail	16 (21.9)	6 (16.2)		16 (22.2)	6 (15.8)	
Liver metastasis	No	26 (35.6)	8 (21.6)	0.200	25 (34.7)	9 (23.7)	0.330
	Yes	47 (64.4)	29 (78.4)		47 (65.3)	29 (76.3)	
Lung metastasis	No	62 (84.9)	33 (89.2)	0.770	61 (84.7)	34 (89.5)	0.571
	Yes	11 (15.1)	4 (10.8)		11 (15.3)	4 (10.5)	
Peritoneal involvement	No	55 (75.3)	29 (78.4)	0.907	55 (76.4)	29 (76.3)	1.000
	Yes	18 (24.7)	8 (21.6)		17 (23.6)	9 (23.7)	
First-line chemotherapy regimen	FOLFIRINOX	48 (65.8)	17 (45.9)	0.042	47 (65.3)	18 (47.4)	0.058
	Nab-paclitaxel + Gemcitabine	12 (16.4)	4 (10.8)		12 (16.7)	4 (10.5)	
	Platinum + Gemcitabine	7 (9.6)	9 (24.3)		7 (9.7)	9 (23.7)	
	Other/unspecified	6 (8.2)	7 (18.9)		6 (8.3)	7 (18.4)	
Treatment response to first-line chemotherapy	Any response	57 (78.1)	14 (37.8)	<0.001	57 (79.2)	14 (36.8)	<0.001
	No response	16 (21.9)	23 (62.2)		15 (20.8)	24 (63.2)	
NLR	Low	34 (46.6)	28 (75.7)	0.004	33 (45.8)	29 (76.3)	0.003
	High	39 (53.4)	9 (24.3)		39 (54.2)	9 (23.7)	
CAR	Low	48 (65.8)	29 (78.4)	0.259	48 (66.7)	28 (73.7)	0.418
	High	25 (34.2)	8 (21.6)		24 (33.3)	10 (26.3)	
LAR	Low	50 (68.5)	26 (70.3)	0.401	49 (68.1)	28 (73.7)	0.611
	High	23 (31.5)	11 (29.7)		23 (31.9)	10 (26.3)	
GINI	Low	42 (57.5)	27 (73.0)	0.069	44 (61.1)	30 (78.9)	0.127
	High	31 (42.5)	10 (27.0)		28 (38.9)	8 (21.1)	

Abbreviations: UAzAR, uric acid z-score-to-albumin ratio; UAzLR, uric acid z-score-to-lymphocyte ratio; ECOG, Eastern Cooperative Oncology Group performance status; NLR, neutrophil-to-lymphocyte ratio; CAR, C-reactive protein-to-albumin ratio; LAR, lactate dehydrogenase-to-albumin ratio; GINI, global immune-nutrition-inflammation index. Cut-offs: UAzAR ≥ 0.0102 and UAzLR ≥ 0.00026. Percentages are calculated using group totals (UAzAR Low *n* = 73, High *n* = 37; UAzLR Low *n* = 72, High *n* = 38). *p* values were calculated using the χ^2^ test or Fisher’s exact test, as appropriate; *p* < 0.001 is reported as <0.001.

**Table 2:** Diagnostic performance of pretreatment indices for 12-month OS and 6-month PFS.

Biomarker	AUC (OS, 12 mo)	*p*	AUC (PFS, 6 mo)	*p*	Youden Cut-Off (OS)	Sensitivity	Specificity
UAzAR	0.659	<0.001	0.705	<0.001	0.0102	0.596	0.879
UAzLR	0.658	<0.001	0.692	<0.001	0.00026	0.596	0.862
NLR	0.716	<0.001	0.675	<0.001	3.33	0.788	0.690
CAR	0.473	0.412	0.572	0.018	1.41	0.750	0.328
LAR	0.584	0.041	0.540	0.214	4.18	0.865	0.397
GINI	0.602	0.008	0.640	<0.001	1077.6	0.788	0.466

Abbreviations: UAzAR, uric acid z-score-to-albumin ratio; UAzLR, uric acid z-score-to-lymphocyte ratio; NLR, neutrophil-to-lymphocyte ratio; CAR, C-reactive protein-to-albumin ratio; LAR, lactate dehydrogenase-to-albumin ratio; GINI, global immune-nutrition-inflammation index. Cutoff values were determined using the Youden index based on 12-month OS ROC analyses and were identically applied to PFS analyses. *p*-values test the null hypothesis that AUC = 0.5 (no discriminative ability).

**Table 3:** Multivariable logistic regression analysis of chemotherapy failure following first-line treatment.

Predictor	Adjusted OR	95% CI	*p*
High UAzAR	5.52	2.16–14.06	<0.001
High UAzLR	6.42	2.49–16.55	<0.001

Abbreviations: UAzAR, sex-adjusted uric acid-to-albumin ratio; UAzLR, sex-adjusted uric acid-to-lymphocyte ratio; OR, odds ratio; CI, confidence interval. Models were adjusted for age (≥65 years), ECOG performance status (0–1 vs. ≥2), presence of liver metastasis at diagnosis, and first-line chemotherapy regimen (FOLFIRINOX vs. non-FOLFIRINOX). Chemotherapy failure was defined as progressive disease (PD) as the best response to first-line chemotherapy. The analysis includes the entire study cohort.

**Table 4:** Univariate Cox regression analyses for overall survival and progression-free survival.

Variable	Comparison	Overall Survival		Progression-Free Survival	
		HR (95% CI)	*p*	HR (95% CI)	*p*
Age	per 1-year increase	1.02 (1.00–1.05)	0.022	1.02 (1.00–1.04)	0.034
ECOG performance status	≥2 vs. 0–1	1.19 (0.77–1.83)	0.428	1.54 (1.01–2.33)	0.043
Diabetes mellitus	Yes vs. No	1.48 (0.97–2.25)	0.069	1.33 (0.88–2.00)	0.175
CAR	High vs. Low	1.70 (1.08–2.66)	0.021	1.94 (1.25–3.00)	0.003
GINI	High vs. Low	2.22 (1.44–3.41)	<0.001	2.76 (1.77–4.30)	<0.001
LAR	High vs. Low	2.01 (1.28–3.16)	0.003	1.50 (0.97–2.30)	0.065
NLR	High vs. Low	3.26 (2.14–4.95)	<0.001	2.98 (1.96–4.54)	<0.001
Treatment response	Any vs. None	0.30 (0.20–0.45)	<0.001	0.21 (0.14–0.33)	<0.001
Chemo regimen	Non-FOLFIRINOX ^†^ vs. FOLFIRINOX	1.57 (0.96–2.58)	0.075	1.58 (0.97–2.58)	0.067
UAzAR	High vs. Low	5.13 (2.63–10.01)	<0.001	3.72 (1.99–6.96)	<0.001
UAzLR	High vs. Low	5.33 (2.74–10.37)	<0.001	3.77 (2.04–7.00)	<0.001

Abbreviations: UAzAR, uric acid z-score-to-albumin ratio; UAzLR, uric acid z-score-to-lymphocyte ratio; ECOG performance status; NLR, neutrophil-to-lymphocyte ratio; CAR, C-reactive protein-to-albumin ratio; LAR, lactate dehydrogenase-to-albumin ratio; GINI, global immune-nutrition-inflammation index; ^†^, nab-paclitaxel plus gemcitabine-based or platinum plus gemcitabine-based regimens. Variables with *p* < 0.10 in the univariate analyses were included in the multivariate analyses. Age and ECOG performance status were forced into all multivariate analyses based on clinical relevance.

**Table 5:** (**A**) Multivariable Cox regression model including UazAR. (**B**) Multivariable Cox regression model including UazLR.

Variable	Overall Survival		Progression-Free Survival	
	HR (95% CI)	*p*	HR (95% CI)	*p*
(**A**)				
Age (per year)	1.01 (0.99–1.03)	0.531	1.00 (0.98–1.02)	0.897
Any treatment response (yes vs. no)	0.43 (0.27–0.69)	<0.001	0.28 (0.17–0.47)	<0.001
ECOG performance status ≥2 vs. 0–1	1.10 (0.68–1.77)	0.694	1.25 (0.77–2.01)	0.369
Diabetes mellitus (yes vs. no)	1.38 (0.87–2.19)	0.166	-	-
NLR high vs. low	1.76 (1.03–3.02)	0.040	1.37 (0.78–2.41)	0.271
Chemo regimen (Nab-paclitaxel + gemcitabine vs. FOLFIRINOX)	1.34 (0.80–2.26)	0.269	1.52 (0.89–2.59)	0.125
Chemo regimen (Platinum + gemcitabine vs. FOLFIRINOX)	-	-	2.38 (1.33–4.28)	0.004
UAzAR high vs. low	3.10 (1.58–6.09)	0.001	2.35 (1.22–4.52)	0.010
(**B**)				
Age (per year)	1.01 (0.99–1.03)	0.514	1.00 (0.98–1.02)	0.894
Any treatment response (yes vs. no)	0.42 (0.27–0.67)	<0.001	0.28 (0.17–0.46)	<0.001
ECOG PS ≥2 vs. 0–1	1.11 (0.69–1.79)	0.671	1.25 (0.78–2.02)	0.358
Diabetes mellitus (yes vs. no)	1.35 (0.85–2.14)	0.201	-	-
NLR high vs. low	1.72 (1.00–2.97)	0.049	1.34 (0.76–2.36)	0.310
Chemo regimen (Nab-paclitaxel + gemcitabine vs. FOLFIRINOX)	1.36 (0.81–2.29)	0.244	1.53 (0.90–2.62)	0.117
Chemo regimen (Platinum + gemcitabine vs. FOLFIRINOX)	-	-	2.41 (1.34–4.34)	0.003
UAzLR high vs. low	3.28 (1.68–6.39)	<0.001	2.47 (1.30–4.70)	0.006

Abbreviations: UAzAR, uric acid z-score-to-albumin ratio; UAzLR, uric acid z-score-to-lymphocyte ratio; ECOG, Eastern Cooperative Oncology Group performance status; NLR, neutrophil-to-lymphocyte ratio. Variables with *p* < 0.10 in the univariate analyses were included; age and ECOG performance status were forced into all models. UAzAR and UAzLR were modeled in separate multivariate models to avoid collinearity.

## Data Availability

The datasets generated and analyzed during the current study are available from the corresponding author upon reasonable request due to ethical and privacy considerations, subject to approval by the Clinical Oncology Department of the University of Health Sciences Antalya Training and Research Hospital.

## Supplementary Materials

The following supporting information can be downloaded at: https://www.mdpi.com/article/10.3390/diagnostics16091296/s1. Table S1: Time-dependent ROC analyses based on inverse probability of censoring weighting (IPCW) were performed to evaluate the discriminative performance of pretreatment biomarkers for overall survival (OS) at multiple clinically relevant time points; Table S2: Time-dependent ROC analyses using IPCW were conducted to assess the discriminative ability of pretreatment biomarkers for progression-free survival (PFS) across early and intermediate follow-up intervals.

## Abbreviations

The following abbreviations are used in this manuscript:

AUC	Area under the curve
CAR	C-reactive protein-to-albumin ratio
CR	Complete response
CRP	C-reactive protein
ECOG	Eastern Cooperative Oncology Group
GINI	Global immune–nutrition–inflammation index
IPCW	Inverse probability of censoring weighting
IQR	Interquartile range
LAR	Lactate dehydrogenase-to-albumin ratio
LDH	Lactate dehydrogenase
NLR	Neutrophil-to-lymphocyte ratio
OS	Overall survival
PD	Progressive disease
PDAC	Pancreatic ductal adenocarcinoma
PFS	Progression-free survival
PR	Partial response
RECIST	Response Evaluation Criteria in Solid Tumors
ROC	Receiver operating characteristic
SD	Stable disease
UA	Uric acid
UAz	Uric acid z-score
UAzAR	Uric acid z-score-to-albumin ratio
UAzLR	Uric acid z-score-to-lymphocyte ratio
