# Supplementary material for: Uric Acid-Driven Biomarkers and Clinical Outcomes in Metastatic Pancreatic Cancer: A Multicenter Real-World Cohort Study
Source: Diagnostics (Basel). 2026 Apr 26;16(9):1296. doi: 10.3390/diagnostics16091296 (PMC13163270; doi:10.3390/diagnostics16091296)
Supplement: Supplementary file 1 [file diagnostics-16-01296-s001.zip › diagnostics-4239377-supplementary.pdf]

Supplementary Table S1.

Time-dependent receiver operating characteristic (ROC) analysis for overall survival

Time-dependent ROC analyses based on inverse probability of censoring weighting (IPCW) were performed to evaluate the discriminative performance of pretreatment biomarkers for overall survival (OS) at multiple clinically relevant time points.

Biomarker	6-month AUC	12-month AUC	18-month AUC	24-month AUC
UAzAR	0.671	0.659	0.602	0.649
UAzLR	0.645	0.654	0.589	0.643
NLR	0.739	0.692	0.725	0.661
GINI	0.733	0.592	0.646	0.604
LAR	0.705	0.584	0.619	0.586
CAR	0.642	0.473	0.539	0.500

Abbreviations: UAzAR, sex-adjusted uric acid z-score–to–albumin ratio; UAzLR, sex-adjusted uric acid z-score–to–lymphocyte ratio; NLR, neutrophil-to-lymphocyte ratio; GINI, global immune-nutrition-inflammation index; LAR, lactate dehydrogenase-to-albumin ratio; CAR, C-reactive protein-to-albumin ratio.

Supplementary Table S2.

Time-dependent receiver operating characteristic (ROC) analysis for progression-free survival

Time-dependent ROC analyses using IPCW were conducted to assess the discriminative ability of pretreatment biomarkers for progression-free survival (PFS) across early and intermediate follow-up intervals.

Biomarker	3-month AUC	6-month AUC	9-month AUC	12-month AUC
UAzAR	0.604	0.705	0.701	0.730
UAzLR	0.587	0.688	0.693	0.716
NLR	0.747	0.652	0.660	0.673
GINI	0.727	0.632	0.638	0.664
CAR	0.627	0.572	0.561	0.557
LAR	0.768	0.540	0.589	0.553

Abbreviations: UAzAR, sex-adjusted uric acid z-score–to–albumin ratio; UAzLR, sex-adjusted uric acid z-score–to–lymphocyte ratio; NLR, neutrophil-to-lymphocyte ratio; GINI, global immune-nutrition-inflammation index; LAR, lactate dehydrogenase-to-albumin ratio; CAR, C-reactive protein-to-albumin ratio.
